# Stratification of malignant renal neoplasms from cystic renal lesions using deep learning and radiomics features based on a stacking ensemble CT machine learning algorithm

**DOI:** 10.3389/fonc.2022.1028577

**Published:** 2022-10-25

**Authors:** Quan-Hao He, Hao Tan, Fang-Tong Liao, Yi-Neng Zheng, Fa-Jin Lv, Qing Jiang, Ming-Zhao Xiao

**Affiliations:** ^1^ Department of Urology, The First Affiliated Hospital of Chongqing Medical University, Chongqing, China; ^2^ Department of Radiology, The First Affiliated Hospital of Chongqing Medical University, Chongqing, China; ^3^ Department of Urology, The Second Affiliated Hospital of Chongqing Medical University, Chongqing, China

**Keywords:** renal neoplasms, machine learning, Bosniak-2019 classification, cystic renal lesions, radiomics analysis

## Abstract

Using nephrographic phase CT images combined with pathology diagnosis, we aim to develop and validate a fusion feature-based stacking ensemble machine learning model to distinguish malignant renal neoplasms from cystic renal lesions (CRLs). This retrospective research includes 166 individuals with CRLs for model training and 47 individuals with CRLs in another institution for model testing. Histopathology results are adopted as diagnosis criterion. Nephrographic phase CT scans are selected to build the fusion feature-based machine learning algorithms. The pretrained 3D-ResNet50 CNN model and radiomics methods are selected to extract deep features and radiomics features, respectively. Fivefold cross-validated least absolute shrinkage and selection operator (LASSO) regression methods are adopted to identify the most discriminative candidate features in the development cohort. Intraclass correlation coefficients and interclass correlation coefficients are employed to evaluate feature’s reproducibility. Pearson correlation coefficients for normal distribution features and Spearman’s rank correlation coefficients for non-normal distribution features are used to eliminate redundant features. After that, stacking ensemble machine learning models are developed in the training cohort. The area under the receiver operator characteristic curve (ROC), calibration curve, and decision curve analysis (DCA) are adopted in the testing cohort to evaluate the performance of each model. The stacking ensemble machine learning algorithm reached excellent diagnostic performance in the testing dataset. The calibration plot shows good stability when using the stacking ensemble model. Net benefits presented by DCA are higher than the Bosniak 2019 version classification when employing any machine learning algorithm. The fusion feature-based machine learning algorithm accurately distinguishes malignant renal neoplasms from CRLs, which outperformed the Bosniak 2019 version classification, and proves to be more applicable for clinical decision-making.

## Introduction

The detection rate of cystic renal lesions (CRLs) is increasing rapidly due to the growing use of computed tomography (CT). However, a considerable number of CRLs are simple renal cysts or benign cystic renal neoplasms according to histopathologic results after surgery; only a proportion of CRLs are malignant renal neoplasms. Cystic renal neoplasms refer to a diverse category of kidney tumors according to the WHO kidney tumor classification, which have a wide range of biological profiles, and the necessity of early surgical treatment for malignant CRL cannot be overstated (1). Since the components of CRL must be identified correctly in order to determine the appropriate treatment strategies, CT imaging is routinely used to distinguish CRLs. Meanwhile, CRLs are difficult to diagnose and manage especially in the early stage as they show a complex pattern in CT images including thickness of septation, enhancement of the mural nodule, and calcifications (2). In an attempt to identify malignant CRL at early stage, standardize the terminology explaining complicated renal cysts, and provide classification standards for stratifying surgically required renal lesions, the Bosniak classification system was created (3). In the 2019 version of the Bosniak classification system, more discriminative and quantitative criteria were introduced to improve the specificity in identifying higher-risk CRL categories and explicit detailed meanings about key terms to promote agreement and consistency among different readers. Based on the updated Bosniak classification, one or more enhancing nodules in CRL with obtuse margins (more than 4 mm) or with acute margins represent malignant renal neoplasms. Thickened wall or septa with enhancement in CRL also suggests the possibility of malignancy (4). However, according to the Bosniak classification, these high-risk CRL (IIF, III, and IV) could still be benign renal cysts rather than malignant neoplasms. Inaccurate treatment and related diagnostic errors caused by the misapplication of Bosniak classification may increase excessive medical care following undesirable outcomes including renal function impairment, re-operation, and neoplastic transplantation (5, 6). It has been shown that the diagnostic performances of the Bosniak 2019 classification criteria do not improve considerably compared with the previous version. A large number of previous Class III lesions will be reclassified as IIF according to the 2019 Bosniak version, resulting in decreased sensitivity (7, 8). The majority of Bosniak I and II lesions are benign renal cysts and Bosniak IIF, III, and IV lesions are more likely to be renal neoplasms. Approximately 10%–20% of Bosniak IIF lesions, 50% of Bosniak III lesions, and 90% of Bosniak IV lesions are considered to be renal neoplasms according to the latest research (9). To increase the diagnosis sensitivity and overcome the limits of biased visual image assessment, quantitative image analysis approaches using machine learning techniques also known as radiomics have gained popularity in recent years (10). In this study, we aim to develop and validate a stacking ensemble-based machine learning model using deep learning and radiomics features to stratify malignant cystic renal neoplasm more precisely.

## Materials and methods

### Participant enrollment and pathology assessment

**Figure 1:**
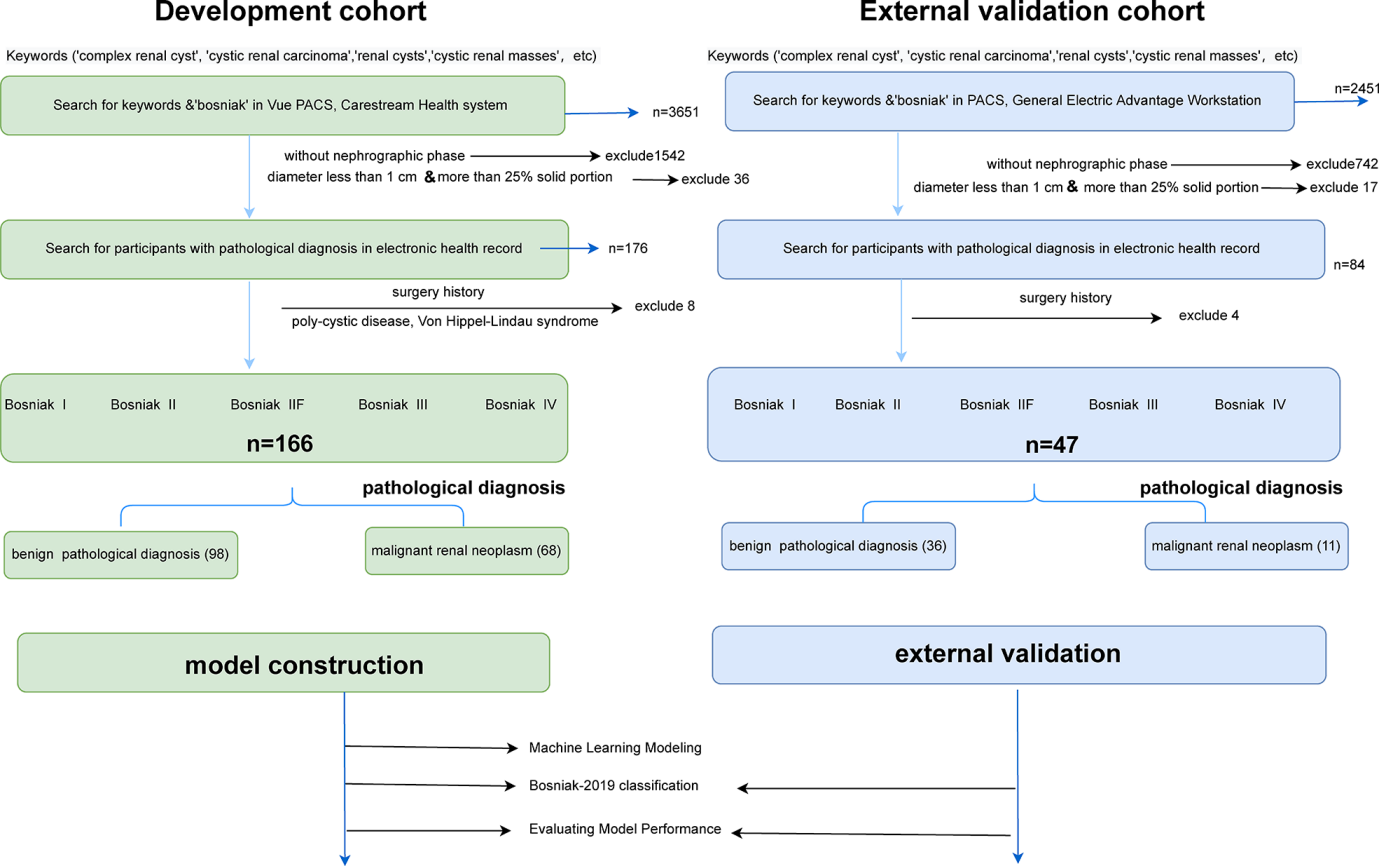
Flowchart illustrating how the cystic renal masses were enrolled and the distribution of pathology results in the final datasets. Detailed inclusion and exclusion criteria are also displayed in the flowchart. CRLs were classified as benign or malignant CRL based on pathological findings. After that, training datasets were applied to build machine learning models, and testing datasets were adopted to reclassify cystic renal masses based on the Bosniak 2019 version and assess model performance.

In this retrospective study, data originated from abdominal CT scans or dedicated CT urography (CTU) scans in two separate institutions comprising unenhanced phases, corticomedullary phases, and nephrographic phases (Vue PACS, Carestream Health Inc & General Electric Advantage Workstation). Ethics committees in both institutions approved this retrospective investigation. Candidate participants included those with renal cysts larger than 1 cm, those with no surgery history (renal needle biopsy, nephrolithotomy, nephrectomy, or partial nephrectomy), those without conditions linked to multiple renal cysts (polycystic disease, Von Hippel–Lindau syndrome, or autosomal dominant polycystic kidney disease), and those with less than 25% solid portion in cystic lesions. Each individual in this study could only include verified cystic renal masses based on the final pathology findings, ensuring a realistic and reliable model’s presentation. The detailed selection process and the pathological results of two cohorts are displayed in **Figure 1**.

### Radiomics feature extraction

For extracting handcrafted radiomics features, radiomics feature class can be divided into three groups (1): first-order statistics, (2) shape features, and (3) second-order features. The image type of radiomics features can be divided into three groups: (1) original, (2) log, and (3) wavelet. Using the standard sample parameters setting provided in the official Pyradiomics YAML file, we extracted 1,231 handcrafted radiomics features in each individual.

### 3DResnet50 feature extraction

**Figure 2:**
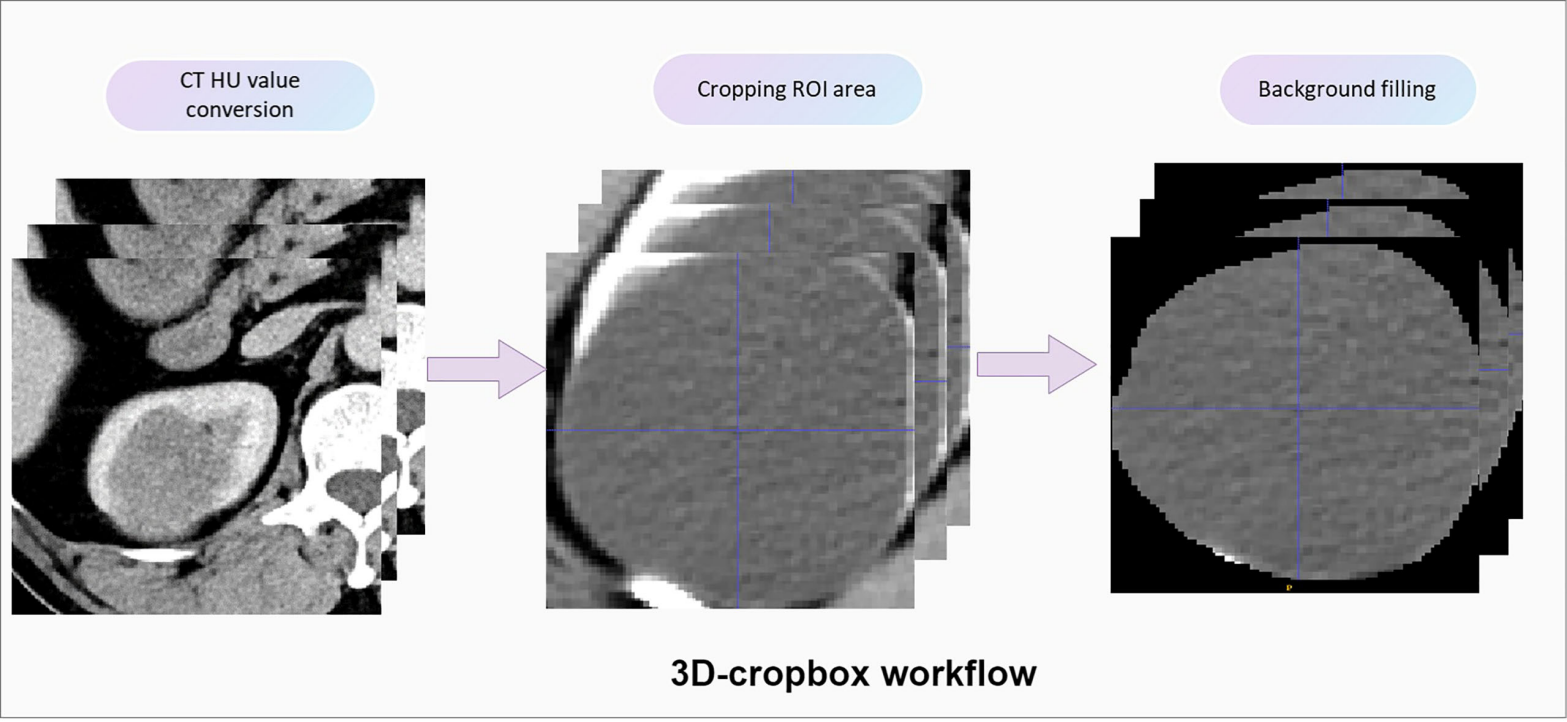
Detailed workflow of the 3D-cropbox. To assure that deep learning features retrieved from the 3D-cropbox are entirely tumor characteristics, the area outside the ROI will be filled with black.

For extracting deep learning features, we defined a 3D-cropbox containing CRL area. The 3D-cropbox’s width and length match the maximum cross-section area of the CRL, while its height matches the dimensions comprising the CRL area. In the 3D-cropbox, the area outside the ROI will be filled with black. After CRL regions have been segmented, the informative slices (the consecutive axial slices containing the full CRL area) will be cropped and resized to 14 mm * 128 mm * 128 mm (14-layer CT scans, default Voxel spacing is 1mm). The preprocessed images will be selected as the input of the convolutional neural network (CNN) model with pretrained weights. Deep learning features in each individual originated from the preprocessed CT images in the ROI area with 14 consecutive slices. **Figure 2** displays the detailed workflow of the 3D-cropbox. By removing the last layer of the pretrained model, disabling gradient updates, and adding a 3D maximum pooling layer, we extracted 2,048 deep learning features in each individual. The detailed 3DResnet50 structure can be found in the **Supplementary Table 1**.

### Radiomics feature harmonization

CT acquisition and reconstruction parameters have a direct impact on handcrafted radiomics features. However, it is impractical to standardize platforms and parameters in advance across different institutions (11). ComBat harmonization, one of the strategies designed to deal with the batch effect, has been widely used in genomics-related research. In this study, ComBat harmonization methods are used to address the difference in extracted radiomics features originating from different image acquisition procedures.

### Correlation coefficient test

The intraclass correlation coefficients and interclass correlation coefficients are utilized to determine selected features with high reproducibility and robustness in image processing setting parameters. The interclass correlation coefficient results are extracted from two independent readers who randomly selected 25% participants in the enrolled datasets. The intraclass correlation coefficient results are calculated from one reader who randomly outlined the same participants in the enrolled datasets at different times (1 month interval) (12).

### Quality control methods

The quality control approach for radiomics analysis and deep learning feature extraction consists of five steps: (1) image quality control, (2) region-of-interest (ROI) quality control, (3) feature extraction quality control, (4) feature selection quality control, and (5) machine learning algorithm quality control. We follow the recommendations from the Image Biomarker Standardization Initiative (IBSI) (13). Radiomics quality scores (RQS) are adopted to assess the reliability in this research (14). Detailed quality control methods and RQS calculation results are introduced in the **Supplementary File**.

### Statistical analysis

**Figure 3:**
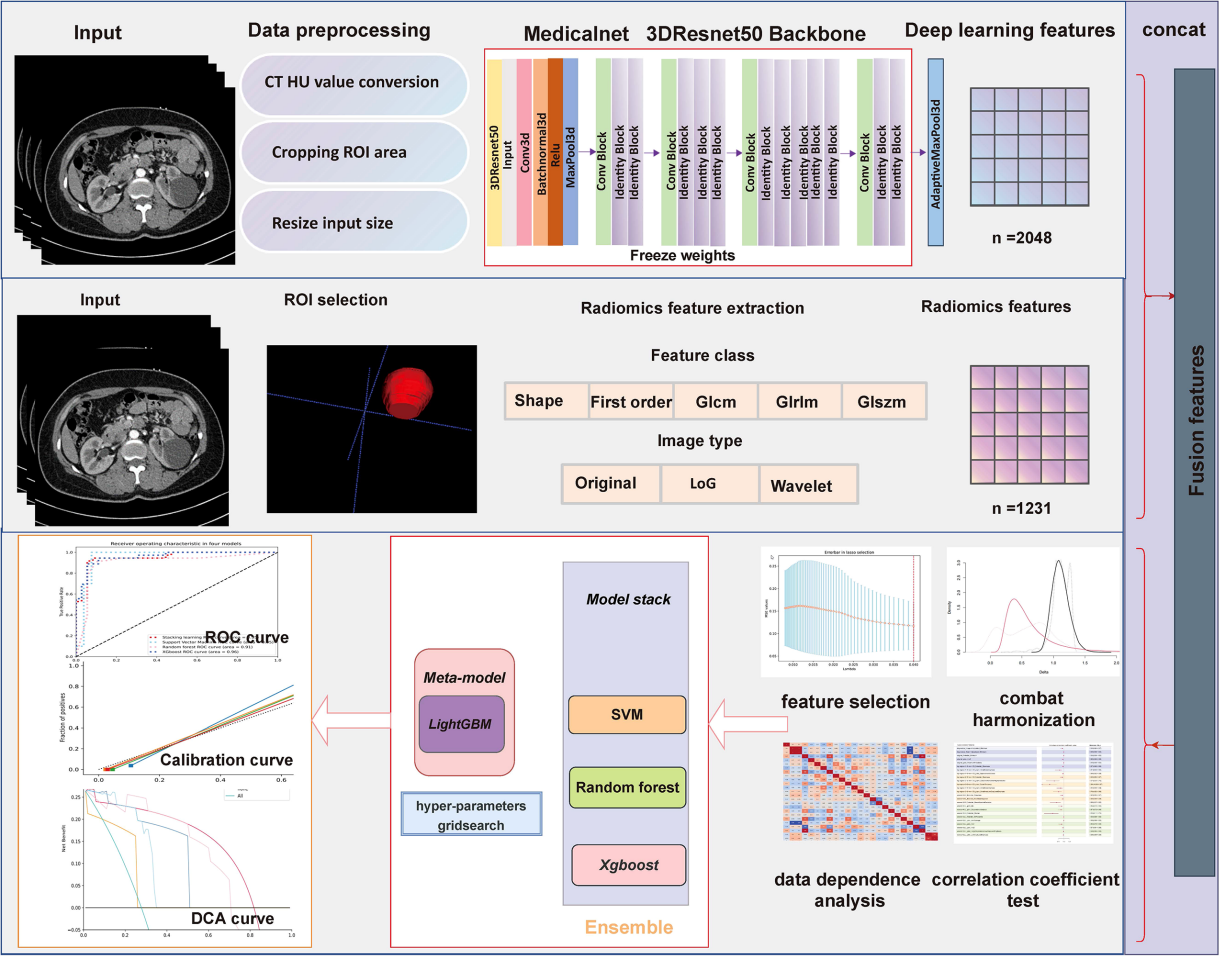
Flowchart presents the detailed procedure in fusion feature-based model construction and assessment methodology.

All ROIs are achieved through ITK-SNAP (version 3.6.0), and radiomics features extraction are executed using Pyradiomics package (version 3.0.1). The pretrained 3DResnet50 model weights come from 23 medical datasets (including brain MR images and lung CT images), which has been an open source published in Tencent’s Medicalnet project (https://github.com/Tencent/MedicalNet). Deep learning features are extracted after adding a 3D maximum pooling layer in the 3DResnet50 model. After feature extraction, the least absolute shrinkage and selection operator (LASSO) approach is selected to choose the most recognized feature subsets in the training datasets (15, 16). We use the fivefold cross-validation method to select candidate variables. Pearson correlation coefficients for normal distribution and Spearman’s rank correlation coefficients for non-normal distribution are used to identify whether redundant features existed in the primary selected radiomic features. Meanwhile, the intraclass correlation coefficients and interclass correlation coefficients are used to assess the final selected feature’s reproducibility. Fivefold cross-validation Grid Search methods are adopted for parameter tuning in the stacking model construction. **Figure 3** depicts the detailed workflow in model construction. Receiver operating characteristic (ROC) curve analysis and accuracy score (ACC) are used to evaluate each model’s performance. DeLong tests are used to evaluate whether the statistical significance of area under the ROC curve (AUC) value exists in four models compared with the Bosniak 2019 version. Calibration curve is adopted to evaluate consistency performances of four models in the testing dataset. Decision curve analysis (DCA) is adopted to assess the clinical practicality. Two-sided *p*-value less than 0.05 is considered statistically significant. All four machine learning models are implemented using the scikit-learn package, and all statistical analysis and plot drawings are implemented using python (3.9 version) and R software (4.0.5 version).

## Results

### Participants

This retrospective study was approved by both hospital ethics committees, and private information of all patients had been de-identified. Among those participants included in development cohort (The First Affiliated Hospital of Chongqing Medical University), 98 individuals were diagnosed as benign CRL and 68 individuals were diagnosed as malignant CRL. In the pathological results of testing cohort (The Second Affiliated Hospital of Chongqing Medical University), 11 individuals have malignant CRL diagnosed and 36 individuals have benign CRL diagnosed. Detailed characteristic distributions are displayed in **Table 1**.

**Table 1:** Detailed distribution of the Bosniak 2019 classification and pathology results in the training cohort and testing cohort.

Bosniak 2019 version		Bosniak I	Bosniak II	Bosniak IIF	Bosniak III	Bosniak IV
	**Pathology analysis**	59	17	23	20	47
	**Benign results**	***n* = 98**				
	Simple renal cysts (89)	59	15	10	5	0
	Cystic nephroma (4)	0	0	1	1	2
	Renal angiomyolipoma (5)	0	0	2	1	2
**Training**	**Malignance results**	***n* = 68**				
**cohort**	Unclassified renal cell carcinoma (5)	0	0	0	0	5
	Clear cell renal cell carcinoma (38)	0	0	3	8	27
	Papillary renal cell carcinoma (10)	0	1	1	1	7
	Chromophobe renal cell carcinoma (3)	0	0	0	1	2
	Multilocular cystic renal neoplasm of low malignant potential (8)	0	0	4	2	2
	Tubulocystic renal cell carcinoma (4)	0	1	2	1	0
	**Pathology analysis**	13	12	9	3	10
	**Benign results**	***n* = 36**				
	Simple renal cysts (34)	13	12	7	2	0
**Testing**	Cystic nephroma (1)	0	0	0	0	1
**cohort**	Mixed epithelial and stromal tumor (1)	0	0	1	0	0
	**Malignance results**	***n* = 11**				
	Clear cell renal cell carcinoma (9)	0	0	0	1	8
	Multilocular cystic renal neoplasm of low malignant potential (2)	0	0	1	0	1

### Machine learning algorithm performance in CRL classification

**Figure 4:**
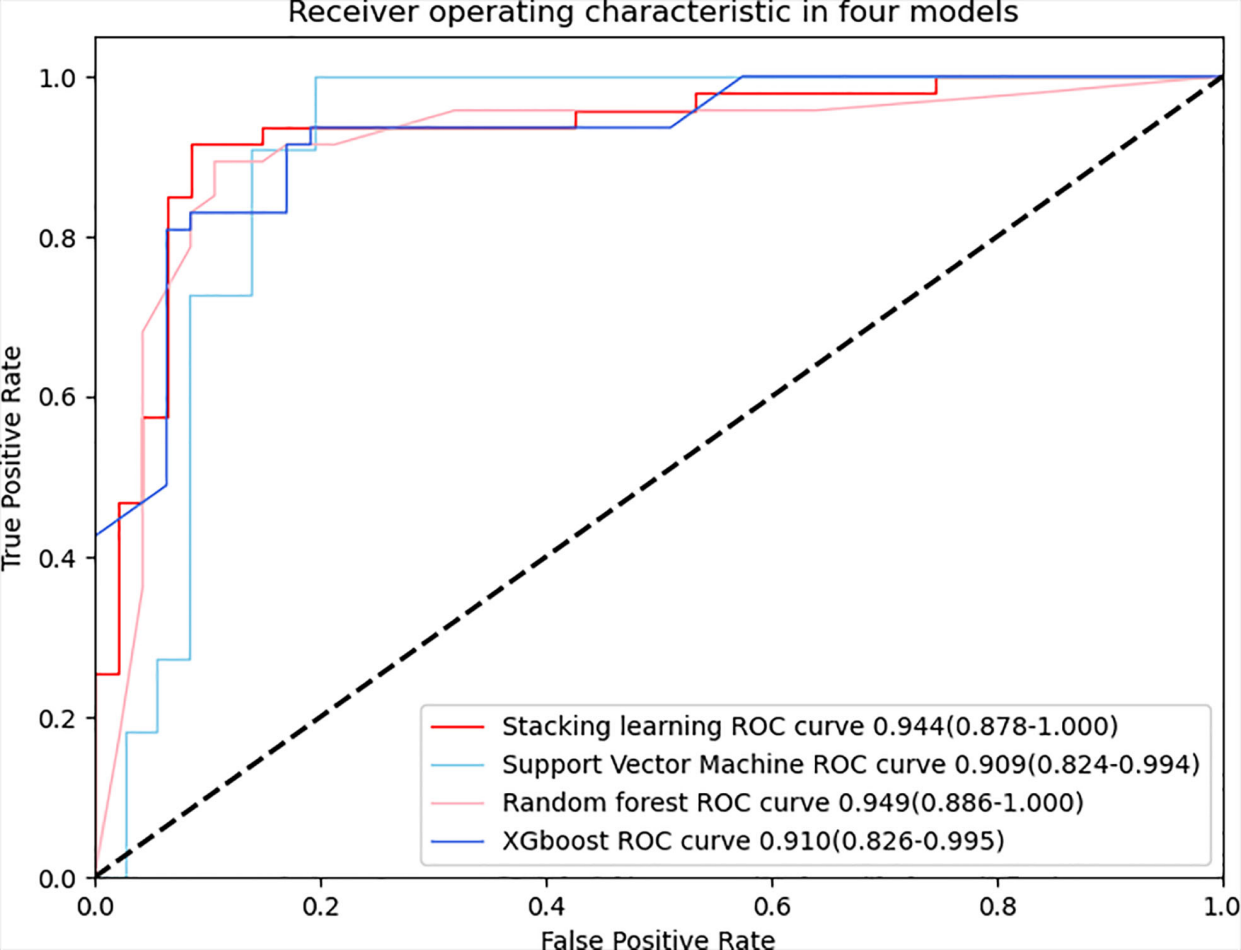
ROC curve is adopted to evaluate the diagnostic efficacy in four models. All four models have similar and excellent performance and detailed AUC values are respectively displayed in the graph, indicating the robust performance of quantitative features after LASSO selection.

**Figure 5:**
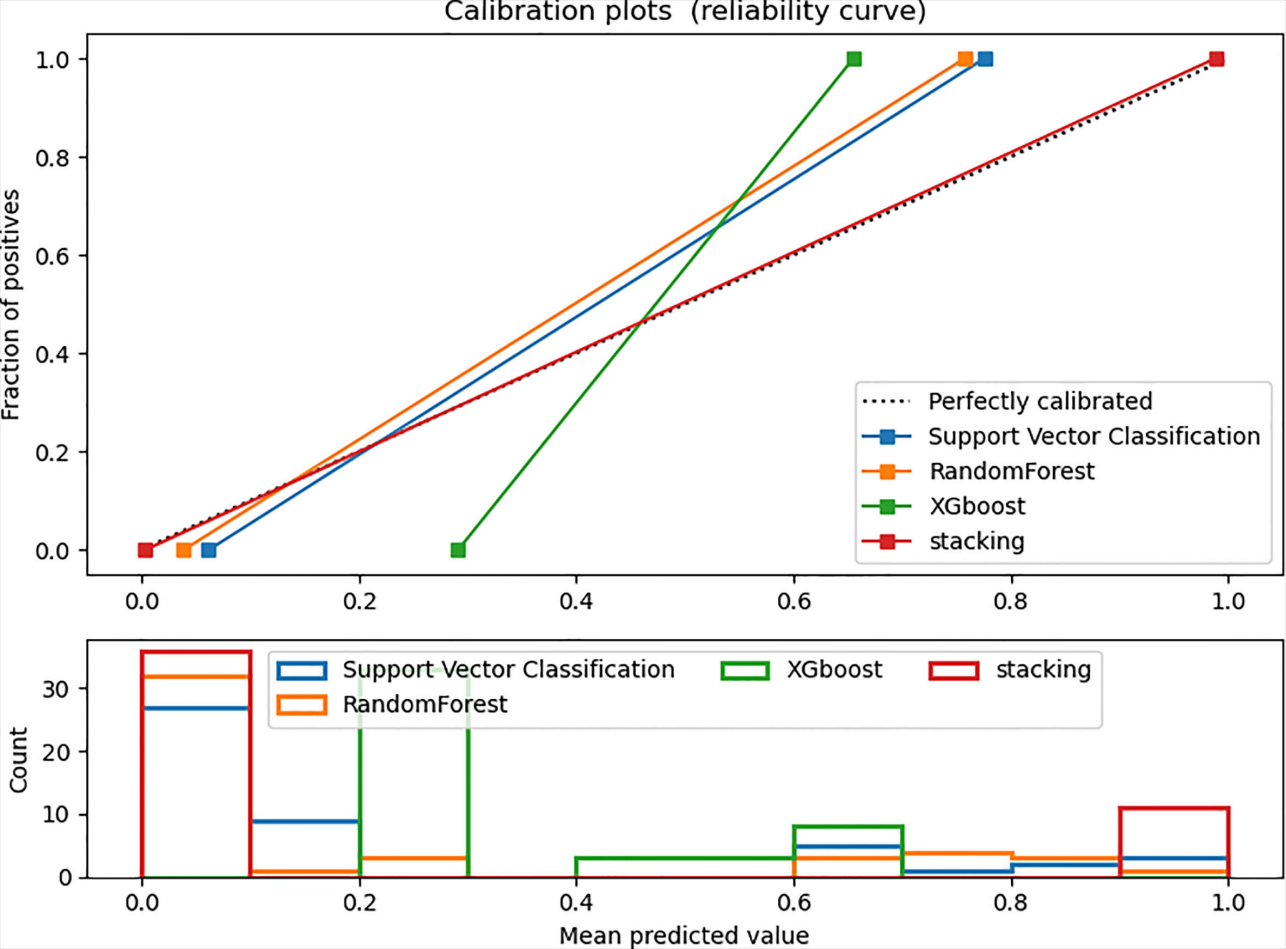
Calibration curve in the testing dataset. The ideal prediction curve is represented by the black dashed line. The model is more accurate as the actual prediction curve gets closer to the dashed line. The bar chart below depicts the average distribution of four models’ predicted probabilities.

**Figures 4** and **5** show the detailed performance of base models and stacking ensemble model, respectively. The AUC values in the base models (Random Forest, Support Vector Machine, Xgboost) and the final model all demonstrate good discriminative ability (**Figure 4**). The *p*-value in the DeLong test shows that the results of the stacking ensemble radiomics model are statistically significant when compared to the Bosniak classification (*p* < 0.05). Detailed performance of four machine learning models compared with the Bosniak 2019 version classification in the training and testing cohorts is displayed in **Table 2**. In **Figure 5**, all four machine learning models illustrate good calibration performance.

**Table 2:** The performance of predictive models and the Bosniak 2019 version classification in the testing cohort and training cohort.

	Model	AUC (95% CI)	ACC (95% CI)	Sensitivity	Specificity	*P*-value in the DeLong test
	Stacking	0.948 (0.912–0.984)	0.916 (0.915–0.917)	0.912 (0.884–0.979)	0.918 (0.864–0.973)	*p* < 0.001
**Train cohort**	Xgboost	0.918 (0.867–0.986)	0.916 (0.915–0.917)	0.882 (0.806–0.959)	0.939 (0.891–0.986)	*p* = 0.051
**(5-fold cross-validation)**	Random forest	0.955 (0.925–0.985)	0.904 (0.903–0.905)	0.926 (0.864–0.989)	0.888 (0.825–0.950)	*p* < 0.001
	Support Vector Machine	0.941 (0.902–0.980)	0.886 (0.884–0.887)	0.956 (0.907–1.000)	0.837 (0.764–0.910)	*p* = 0.002
	Bosniak 2019 classification	0.863 (0.816–0.910)	0.843 (0.842–0.845)	0.971 (0.930–1.000)	0.755 (0.670–0.840)	**ref**
	Stacking	0.944 (0.878–1.000)	0.936 (0.934–0.939)	1.000 (1.000–1.000)	0.917 (0.826–1.000)	*p* = 0.014
	Xgboost	0.910 (0.826–0.995)	0.851 (0.846–0.856)	1.000 (1.000–1.000)	0.806 (0.676–0.935)	*p* = 0.168
**Test cohort**	Random forest	0.949 (0.886–1.000)	0.915 (0.912–0.918)	1.000 (1.000–1.000)	0.889 (0.786–0.992)	*p* = 0.010
	Support Vector Machine	0.909 (0.824–0.994)	0.851 (0.846–0.856)	1.000 (1.000–1.000)	0.806 (0.676–0.935)	*p* = 0.128
	Bosniak 2019 classification	0.847 (0.771–0.924)	0.766 (0.758–0.773)	1.000 (1.000–1.000)	0.694 (0.544–0.845)	**ref**

AUC, area under the receiver operating characteristic curve; ACC, accuracy score; ref, reference in AUC DeLong test, 95%CI; 95% confidence interval.

### Clinical impact of the machine learning model compared with the Bosniak 2019 classification

**Figure 6:**
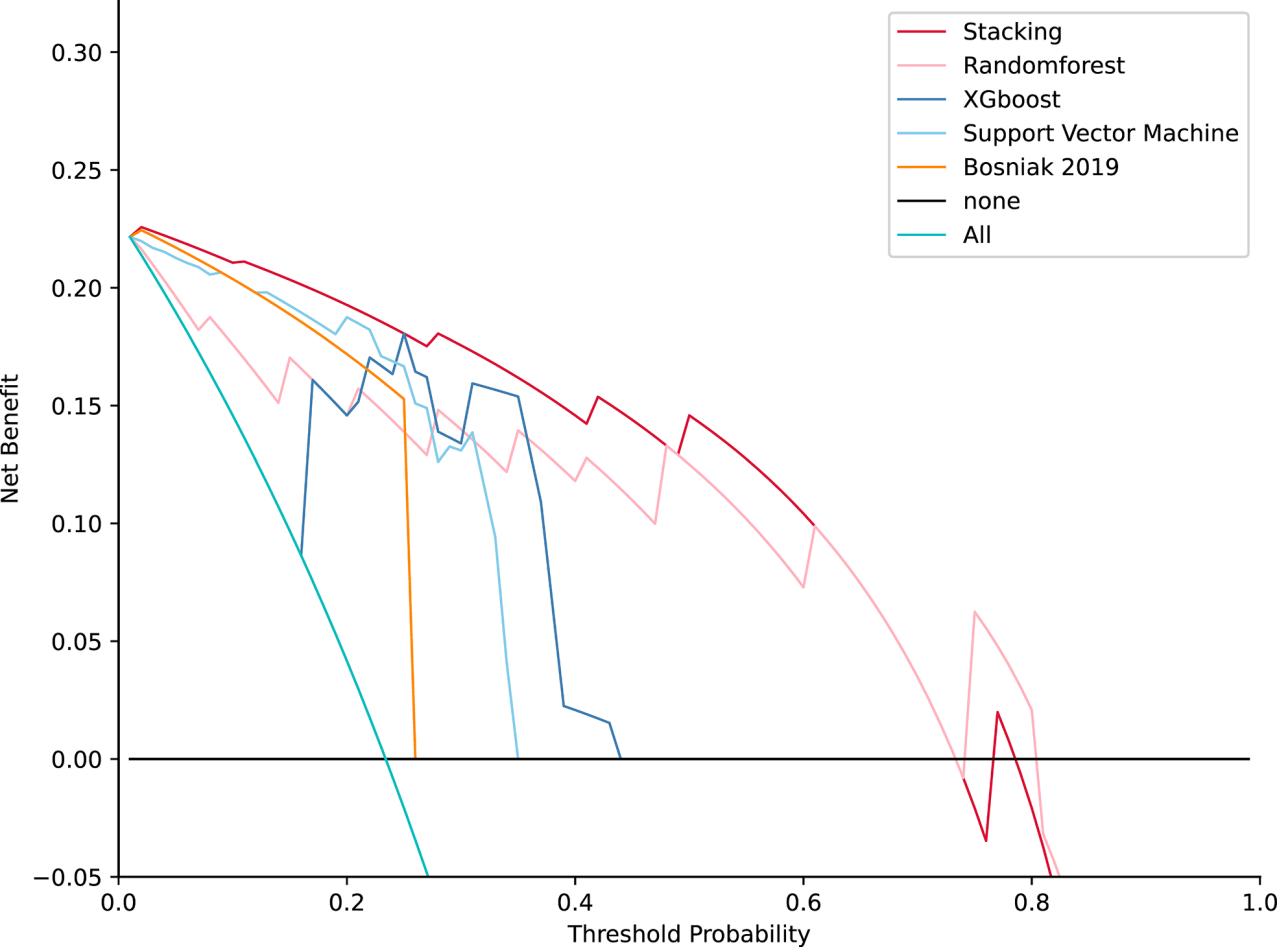
Decision curve analysis for four models compared with the Bosniak 2019 version in testing datasets. The net benefit is represented on the *y*-axis and the corresponding threshold probability is represented on the *x*-axis. The stacking model is represented by the red line. The Bosniak 2019 version is represented by the yellow line. All machine learning models present better performance and offered more net benefit than the Bosniak model.

**Figure 7:**
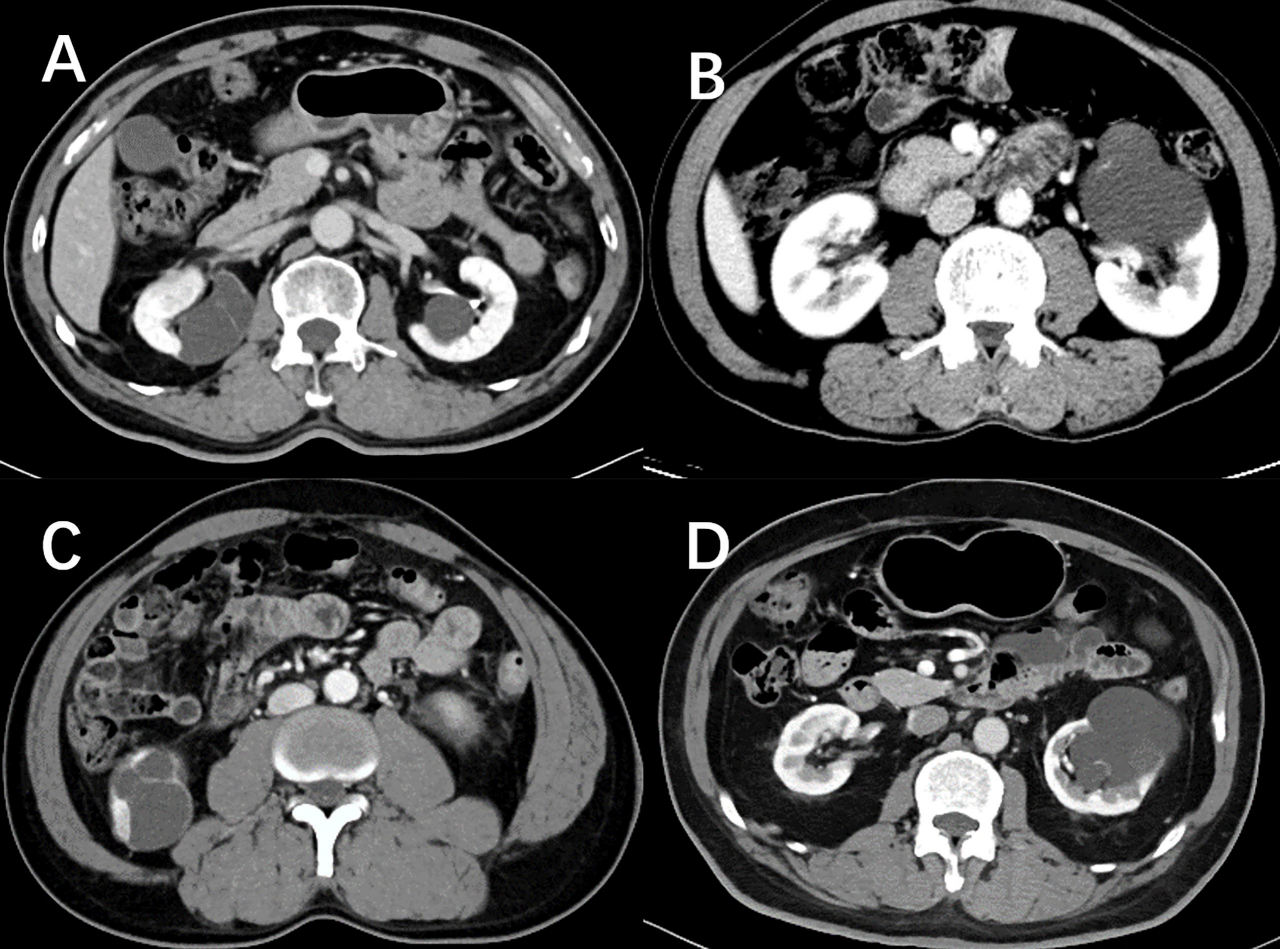
Nephrographic phase images for four cystic renal lesions in the testing datasets. Cystic renal lesion in **(A)** is benign 2019 Bosniak II lesion and cystic renal lesion in **(C)** is identified as clear cell renal cell carcinoma according to the histopathologic results after surgery, which stacking model properly predicted as cystic renal neoplasms. Cystic renal lesion in **(B, D)** are classified as 2019 Bosniak IIF, which are accurately identified as benign CRL by the stacking machine learning model.

DCA for four machine learning models in the testing dataset reveal that all four machine learning models deliver a higher net benefit than “none” and “all” treatment methods under most threshold probabilities (**Figure 6**). Meanwhile, according to the histopathologic results in the testing dataset, all four machine learning models provide a higher net benefit than the management guideline based on the Bosniak classification in terms of correctly stratifying cyst lesions into malignant renal neoplasms and benign renal masses, implying that using machine learning algorithm will provide better clinical decision support. Four instances of the final model’s performance in the testing cohort are presented in **Figure 7**. Detailed confusion matrixes for four machine learning models and the Bosniak classification are displayed in **Supplementary Material**.

## Discussion

Although the updated Bosniak classification system has a close correlation with cystic renal tumors, it has limits when analyzing the pathological results of Bosniak IIF, III, and IV classified CRLs, leading to inappropriate surgical operations and excessive follow-up costs. Identifying low-risk Bosniak classified CRLs can help prevent overtreatment and an increase in healthcare costs (17, 18). Previous findings suggested that the progression of Bosniak IIF cystic renal masses was 4 years (19). The high-risk Bosniak CRL had a quick progression that required radical nephrectomy rather than inappropriate surgical procedures like renal cyst decortication (20, 21). In this retrospective study, using stacking ensemble machine learning methods, we achieved excellent diagnostic performance in discriminating benign CRLs from malignant CRLs, which outperformed the Bosniak classification system. In the final model, we included 19 reproducible and discriminating radiomics features and 5 deep learning features, which displayed robust and consistent performance between cross-validation datasets and testing datasets.

The following elements contribute to the stacking ensemble model’s reliability (1): The research procedure adheres to IBSI guidelines. (2) By using histopathologic examinations as diagnostic criterion, the diagnostic accuracy in this study is confirmed. (3) A stacking ensemble machine learning algorithm prevents overfitting in the training dataset. (4) In the testing step, the stacking model shows good diagnostic performance. (5) The RQS analysis results of this study was 16, which indicated that the quality of this radiomics study was reliable and reproducible. The points were accumulated by adhering to the following criteria: image protocol quality compliance (+1), feature reduction or adjustment for multiple testing compliance (+3), discrimination method with resampling method compliance (+2), calibration statistics method compliance (+1), validation from another institute compliance (+3), comparison to “gold standard” compliance (+2), potential clinical utility (+2), open-sourced code (+1), and open-sourced radiomics features compliance (+1). The updated 2019 version of the Bosniak classification intends to address inter-reader variability and improve diagnostic performance in predicting malignancy CRL. However, the proposed classification ability has yet to be confirmed (22). In this study, two different well-trained readers still disagreed on some CRL issues. In contrast, the stacking decision algorithm demonstrated strong and consistent performance without requiring subjective judgment across the testing datasets.

Many earlier studies have shown that machine learning approaches can be used to stratify CRL (23, 24). However, only a few studies rely on pathology as the diagnostic criteria (25). Miskin et al. used a CT texture-based machine learning method to distinguish CRLs as benign cysts and potentially malignant cysts based on cystic renal mass reclassification using the Bosniak 2019 version (26). However, the Bosniak classification does not have a precise pathological standard, and benign lesions may still be present in these potentially malignant groups, which limited the clinical value. Recently, Reinhold et al. used a CT-based radiomics model with a clinical decision algorithm to distinguish malignant CRLs from CRLs (27). However, they defined benign CRLs as non-imaging changes over 4 years’ follow-up rather than pathological diagnostic criteria that could lead to actual biases, and the ability to distinguish benign from malignant CRLs remains debatable since benign CRLs were not defined by a pathological standard. In this study, all included CRLs have postoperative pathological results, which ensured that the model performance was reliable. Stacking algorithms demonstrated high specificity and sensibility, which may potentially impact clinical practice when radiologists or urologists try to evaluate and determine the right surgical procedure for CRLs.

Although the final machine learning model effectively predicted the outcome of CRL pathology, several limitations should also be mentioned. First, limited by the clinical sample size of CRLs, the diagnostic performance of our machine learning model in large samples still needs to be validated while testing datasets were used in this work. Second, we adopted nephrographic phase CT images to build the machine learning algorithm rather than using triple-phase CT images. In the Bosniak 2019 version, MRI standard features were formally added, whereas there is very little research focusing on renal cysts’ textural features in MR images (28, 29). Previous studies have shown that using renal mass protocol MRI (with subtraction images) can help identify whether there are underlying enhancing features related to malignancy (2, 30). Nephrographic phase CT images and corticomedullary phase CT images for the radiomics model all demonstrated good discriminatory capability when compared with the Bosniak 2019 version categories (31). Future studies could attempt to integrate the triple-phase CT images and MR images by sequence-to-sequence models like the recurrent neural network (RNN) and vision transformer (VIT) (32, 33). Meanwhile, cystic nephroma is typically prevalent in female patients aged 50 to 60 years, which indicates that clinical variables such as age and gender may be potential predictors, and a mixture model that combines radiomics features with clinical features may further improve diagnostic performance (34).

## Conclusion

In summary, a stacking fusion feature-based machine learning meta model demonstrates good discrimination capability in stratifying malignant cystic renal neoplasms in CRLs across the testing datasets, which will be beneficial in diagnosing malignant CRLs at a curable stage, reducing overdiagnosis and overtreatment in CRLs.

## Data availability statement

The raw data supporting the conclusions of this article will be made available by the authors, without undue reservation.

## Ethics statement

Written informed consent was not obtained from the individual(s) for the publication of any potentially identifiable images or data included in this article.

## Author contributions

Q-HH and HT contributed equally to this work and share the first authorship. Q-HH, HT, F-JL, and Y-NZ designed the study; Q-HH, HT, and F-TL performed the experiments and collected the data. Q-HH analyzed the data and wrote the manuscript. M-ZX and QJ reviewed and edited the manuscript. All authors read and approved the final manuscript.

## Funding

This study was supported by the National Key Research and Development Project, No. 2020YFC2005900.

## Acknowledgments

We appreciate all radiologists and related staff in the First Affiliated Hospital of Chongqing Medical University for their assistance in data collection and ROI sketching.

## Conflict of interest

The authors declare that the research was conducted in the absence of any commercial or financial relationships that could be construed as a potential conflict of interest.

## Publisher’s note

All claims expressed in this article are solely those of the authors and do not necessarily represent those of their affiliated organizations, or those of the publisher, the editors and the reviewers. Any product that may be evaluated in this article, or claim that may be made by its manufacturer, is not guaranteed or endorsed by the publisher.
