# Supplementary material for: Stratification of malignant renal neoplasms from cystic renal lesions using deep learning and radiomics features based on a stacking ensemble CT machine learning algorithm
Source: Front Oncol. 2022 Oct 25;12:1028577. doi: 10.3389/fonc.2022.1028577 (PMC9640984; doi:10.3389/fonc.2022.1028577)
Supplement: Supplementary file 1 [file DataSheet_1.docx]

Supplementary material

Detailed enrollment procedure and quality control methods

**Supplementary figure1:** Forest plot shows the ICC coefficients for the final selected features

**Supplementary figure2:** The prior distribution of scale parameters (Delta) before and after handcrafted radiomics feature harmonization

**Supplementary figure3:** The prior distribution of scale parameters (Gamma) before and after handcrafted radiomics feature harmonization

**Supplementary figure4**: The error bar plot in lasso selection

**Supplementary figure5** selected features weights after lasso selection

**Supplementary figure6:** The heatmap for selected features after LASSO regression and ICC analysis.

**Supplementary figure7:** The confusion matrix for four machine learning model and Bosniak-classification in testing datasets

**Supplementary figure8:** The ROC curve of final model in the training cohort using 5-fold cross-validation

**Supplementary figure9:** Circus plot illustrating the corresponding relationship between radiomics features and 3DResnet50 features after lasso selection

**Supplementary table1:** Detailed structure components and parameters in 3D-resnet50 model

Detailed enrollment procedure and quality control methods

Candidate participants enrollment procedure

In this study, all image data in development cohort (The First Affiliated Hospital of Chongqing Medical University) are obtained from picture archiving and communication systems (PACS: RISGC version3.1s19.5, Carestream health Inc.). All image data in testing cohort (The Second Affiliated Hospital of Chongqing Medical University) are obtained from General Electric Advantage Workstation. Corresponding pathology results of CRL are obtained from Computer-based Patient Record (EMR) and electric pathology system in each hospital. In PACS system, keywords associated with CRL (cystic renal masses, cystic renal carcinoma, complex renal cysts, Cystic renal tumor, Bosniak, etc.) are used to initially select images data. The exclusion criteria included images without nephrographic phase, diameter of CRL less than 1cm, more than 25% solid portions. After that, we will search pathology results from EMR and electric pathology system according to the patient information from PACS system. The exclusion criteria in these steps included patients with renal surgery history, poly-cystic disease, and Von Hippel-Lindau syndrome.

quality control methods

Images quality control

In training dataset, Contrast-enhanced CT scans are obtained from 128-slice spiral CT scanner (Siemens Healthcare, Germany) or 64-slice spiral CT scanner (General Electric, USA). In testing dataset, enrolled patients underwent contrast-enhanced imaging with a 128-slice scanner (LightSpeed VCT, GE Medical systems, USA). The standardized protocols in CT image scanning are as follows: Each patient will be given to a three-phase CT scans (plain phase, arterial phase, and venous phase). CT scanning parameters including CT-tube voltage (120-140 kv), CT-tube current (125-300 mAs), scanning matrix (512*512 pixels) and slice thickness (ranging from 1mm to 5mm). After intravenous administration of iohexol (300 mg/mL at a rate of 3.0 mL/s, followed by a 30-mL saline flush), contrast-enhanced CT samples will be captured. The total contrast volume for each kilogram was 1.5 ml. After unenhanced CT scanning, a contrast-enhanced CT scan will be performed. The nephrographic (100-120s) phase images will be selected to do further research.

ROI regions quality control

We follow the ISBI recommendations for verifying the repeatability of ROI regions, which is necessary since the ROI delineation is subjected to significant variation between observers, which may cause the unstable results. Feature reproducibility is assessed by intra-class correlation coefficients and inter-class correlation coefficients (ICC), which are created by comparing features extracted from two independent ROI sketchers. ICC value >0.75 is considered as robust features in previous studies(1, 2). In this study, we select features with ICC values >0.75 as final variables and all 24 radiomics features in the training datasets will be normalized prior to feature selection according to their mean value and standard deviation value (z-score normalization) to make sure the comparability of each selected variable (**Supplementary figure1)**.

Feature extraction quality control

**Data preprocessing for deep learning features**

With the segmentation of the tumor region delineated, the informative slices (the consecutive axial slices containing full tumor area) will be cropped and resized to 14 mm *128mm * 128 mm (the size for the input layer of the 3Dresnet models). The cropped images will be selected as the input of convolutional neural network (CNN) model.

**3Dresnet model architecture**

In our study, Tencent medicalnet 3Dresnet model was adopted for extraction of deep learning features, which was pretrained on 23 medical datasets (including brain MR images and lung CT images, etc.) This model is publicly assessable as open-source code (<https://github.com/Tencent/MedicalNet> ). After data pre-processing for deep learning features and model modification, the cropped images will be propagated in the network to generate deep learning features.

**Handcrafted radiomics Feature definitions**

Handcrafted radiomics features are computed from the radiologist-drawn ROIs using an open-source python package Pyradiomics. Detailed calculations of handcrafted radiomics features are described and provided in online documentation of Pyradiomics (<https://pyradiomics.readthedocs.io/en/latest/features.html> ). We start feature extraction by using the standard sample parameters setting provided in the official Pyradiomics YAML file and all the images will be resampled to 1×1×1 mm³ voxels to standardize the slice thickness.(3). Image intensities are binned by 25 HU and voxel array shift is set on 1000. All radiomics features adopted in this study are in accordance with feature definitions as described by the Imaging Biomarker Standardization Initiative (IBSI).

**Handcrafted radiomics feature harmonization**

Combat methods are used to minimize the multicenter effect caused by different CT scanners and protocol parameters. According to the statistical distribution of the dataset, the nonparametric form model is adopted in Combat methods to determine the transformation for each feature separately using “sva” R package ( <https://bioconductor.org/packages/release/bioc/html/sva.html> ). **Supplementary figure2 and figure3** show the prior distribution of scale parameters (Gamma and Delta) before and after handcrafted radiomics feature harmonization (4).

Feature selection quality control

All regions of interest (ROI) are achieved through ITK-SANP (version 3.6.0) and radiomic features extraction are conducted using Pyradiomics package (version 3.0.1) in python environment (version 3.9 & version 3.7)(5). 1231 radiomics features and 2048 deep learning features are generated in each individual at the beginning. In the development cohort, the least absolute shrinkage and selection operator (LASSO) methods which could add the penalty for non-zero coefficients to the sum of the absolute value (L1 penalty) are selected to filter the candidate variables. All candidate variables will be normalized before LASSO selection. At the selected λ value of 0.022, 34 candidate features are selected by LASSO methods (**supplementary figure4**). The Spearman’s correlation coefficient for non-normal distribution variables and Pearson correlation coefficients for normal distribution variables are employed separately to reduce redundancy between the 34 filtered radiomics features. After Spearman’s correlation coefficient test and Pearson correlation coefficients test, **supplementary figure5** displays the final selected features weights after lasso selection and **supplementary figure6** demonstrates well reproducibility and low correlations in the final 24 selected variables.

Machine learning algorithms quality control

Stacking, also known as stacked generalization, is a strategy for training a meta-model to intelligently combine the predictions of numerous base-models, allowing to reap the benefits of multiple models by integrating their separate predictions with higher-level model(6). The base-models in this study were Random Forest (RF), Support Vector Classification (SVM) and Xgboost. We train the base models on the training datasets and generated corresponding predictions. Next, we combine the predictions of each base models as additional features. In the final step, we train the final meta-model in the training datasets combined with additional features. All base models and stacking model are tested in the testing datasets.

Detailed radiomics quality score (RQS) of this study using RQS1.0 version

The radiomics quality scores (RQS) of this study reach 16. The points cumulated are obtained by complying with image protocol quality (+1), feature reduction or adjustment for multiple testing (+3), discrimination method with resampling method (+2), calibration statistics method (+1), validation from another institute (+3), comparison to “gold standard” (+2), potential clinical utility (+2),

open-sourced code (+1), and open-sourced radiomics features (+1).

Correctness of each model and the confusion matrix in the stacking model

All four models illustrate well performance and satisfactory accuracy score (SVM ACC=85.1%, RF ACC=91.5%, Xgboost ACC=85.1%, Stacking algorithm ACC =93.6%). **Supplementary figure7** shows the confusion matrix for the four machine-learning model and Bosniak-2019 classification in testing datasets.

References

1. Graumann O, Osther SS, Karstoft J, Hørlyck A, Osther PJ. Bosniak Classification System: Inter-Observer and Intra-Observer Agreement among Experienced Uroradiologists. *Acta radiologica (Stockholm, Sweden : 1987)* (2015) 56(3):374-83. Epub 2014/04/01. doi: 10.1177/0284185114529562.

2. Bartko J. The Intraclass Correlation Coefficient as a Measure of Reliability. *Psycholog Rep* (1966) 19.

3. van Griethuysen JJM, Fedorov A, Parmar C, Hosny A, Aucoin N, Narayan V, et al. Computational Radiomics System to Decode the Radiographic Phenotype. *Cancer Research* (2017) 77(21):e104-e7. doi: 10.1158/0008-5472.Can-17-0339.

4. Leek JT, Johnson WE, Parker HS, Jaffe AE, Storey JD. The Sva Package for Removing Batch Effects and Other Unwanted Variation in High-Throughput Experiments. *Bioinformatics (Oxford, England)* (2012) 28(6):882-3. Epub 2012/01/20. doi: 10.1093/bioinformatics/bts034.

5. Yushkevich PA, Pashchinskiy A, Oguz I, Mohan S, Schmitt JE, Stein JM, et al. User-Guided Segmentation of Multi-Modality Medical Imaging Datasets with Itk-Snap. *Neuroinformatics* (2019) 17(1):83-102. Epub 2018/06/28. doi: 10.1007/s12021-018-9385-x.

6. Rajaraman S, Zamzmi G, Antani SK. Novel Loss Functions for Ensemble-Based Medical Image Classification. *PloS one* (2021) 16(12):e0261307. Epub 2021/12/31. doi: 10.1371/journal.pone.0261307.

**Supplementary figure1:** Forest plot shows the ICC coefficients for the final selected 19 radiomics features and 5 deep learning features in the develop cohort. ICCs: Intraclass Correlation Coefficients and interclass correlation coefficients. Inclusion criteria in this study is ICC value greater than 0.75


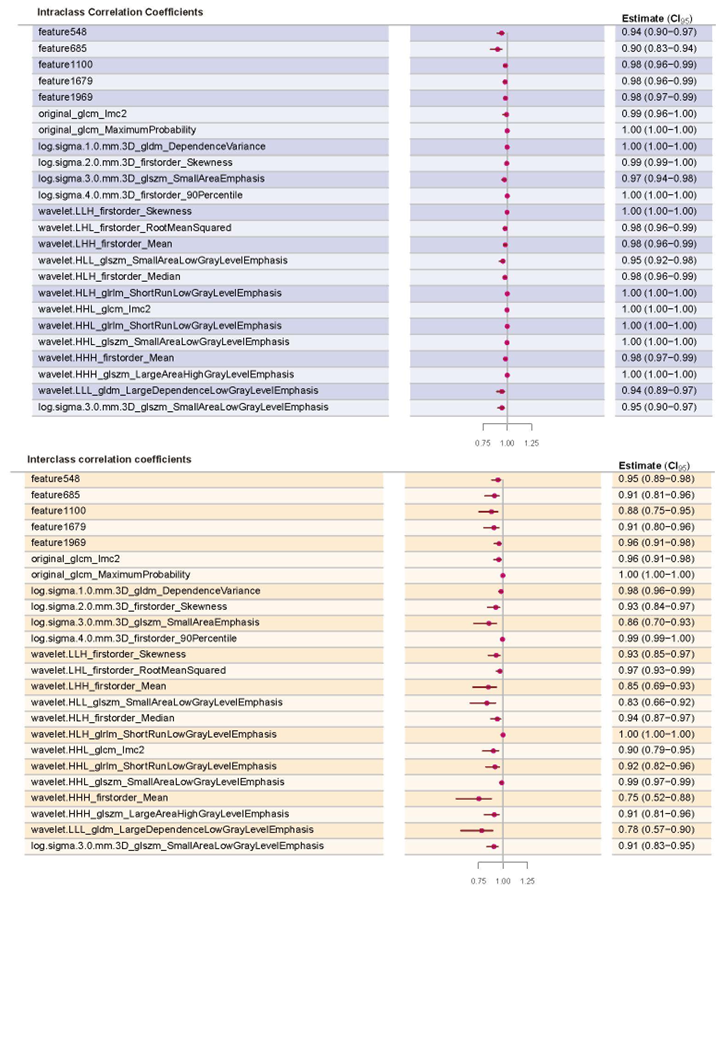


**Supplementary figure2:** The prior distribution of scale parameters (Delta) before and after handcrafted radiomics feature harmonization


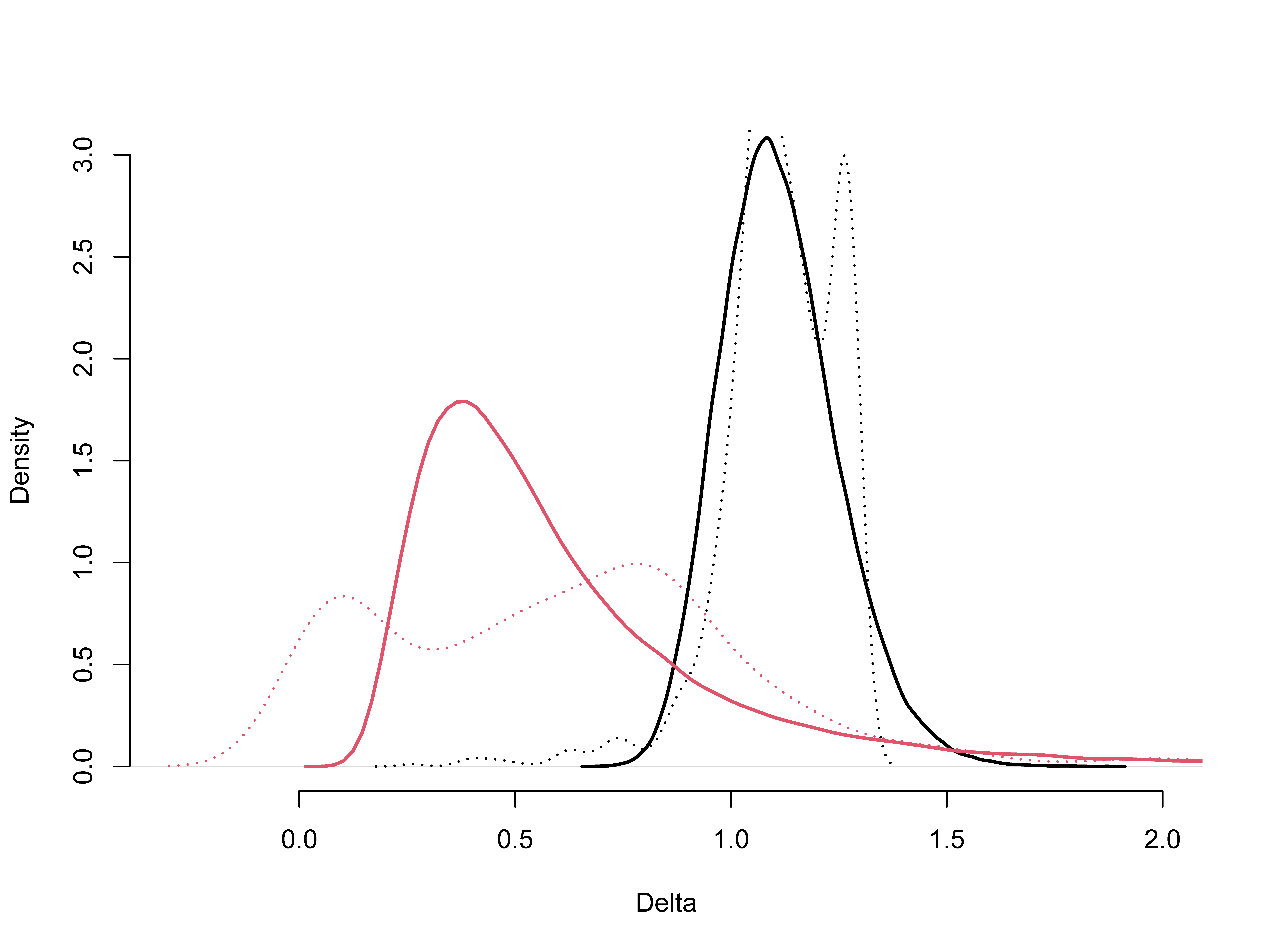


**Supplementary figure3:** The prior distribution of scale parameters (Gamma) before and after handcrafted radiomics feature harmonization


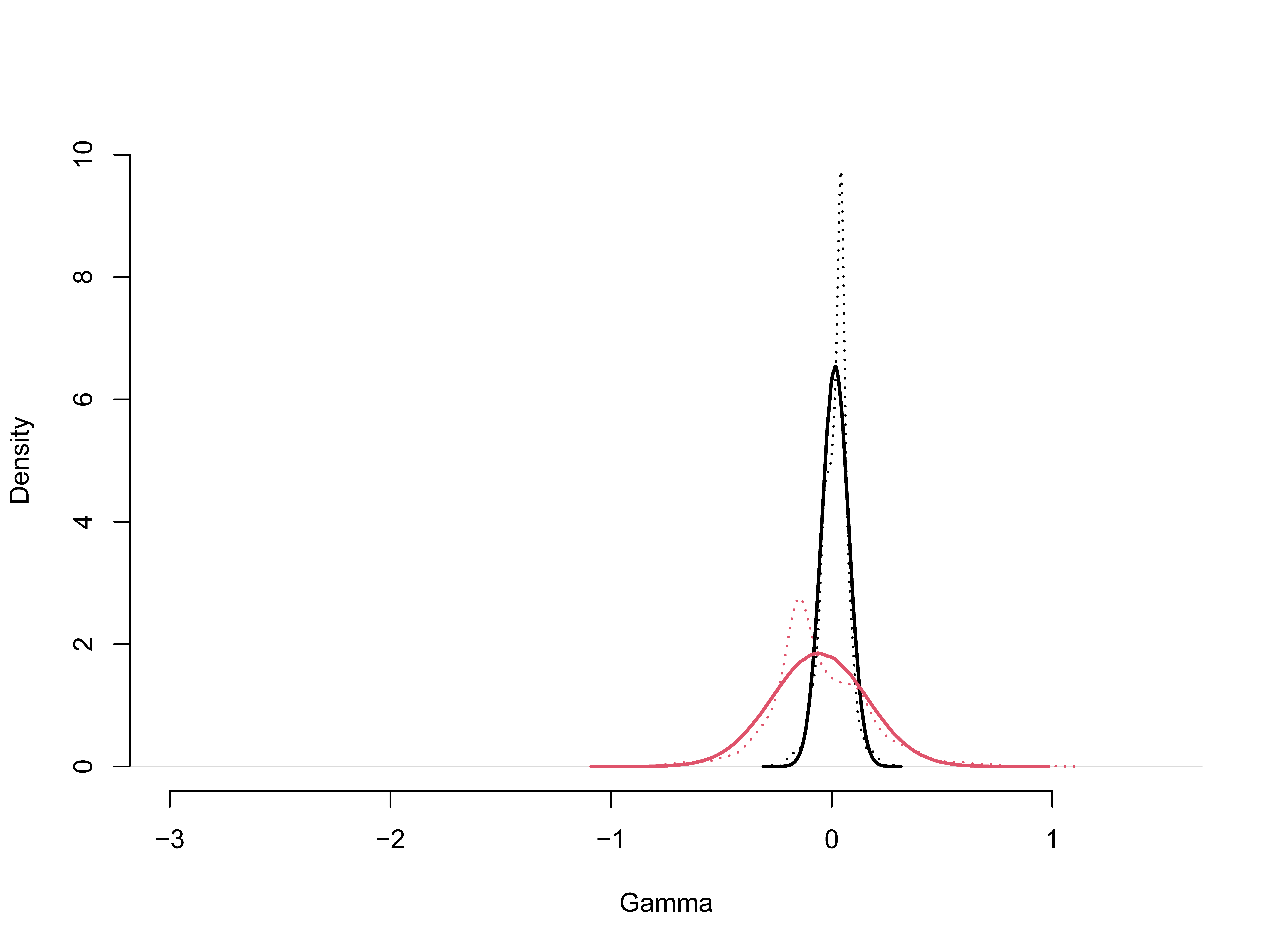


**Supplementary figure4**: The error bar plot in lasso selection and the selected λ value is 0.022


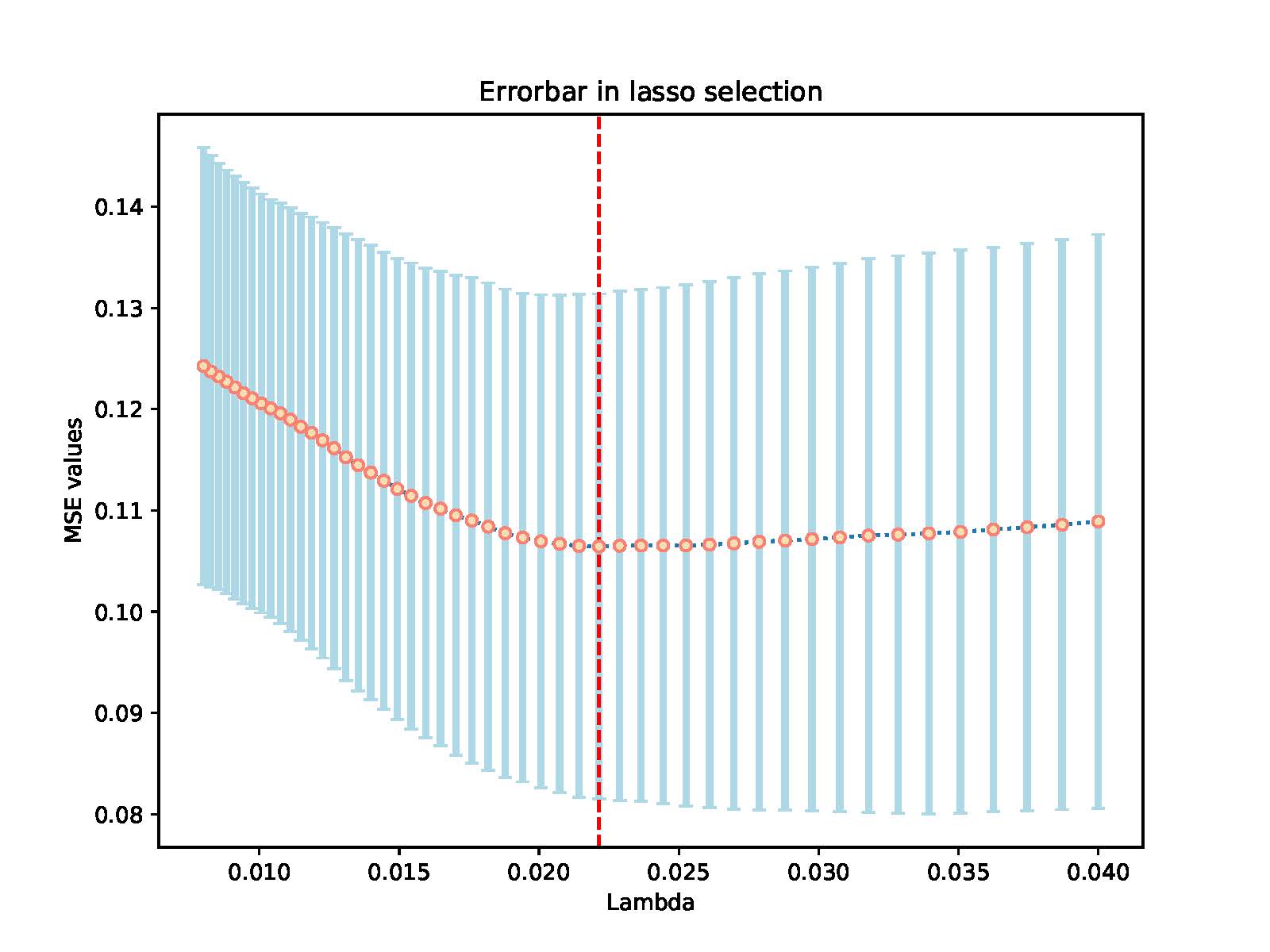


**Supplementary figure5**: Selected features weights after lasso selection


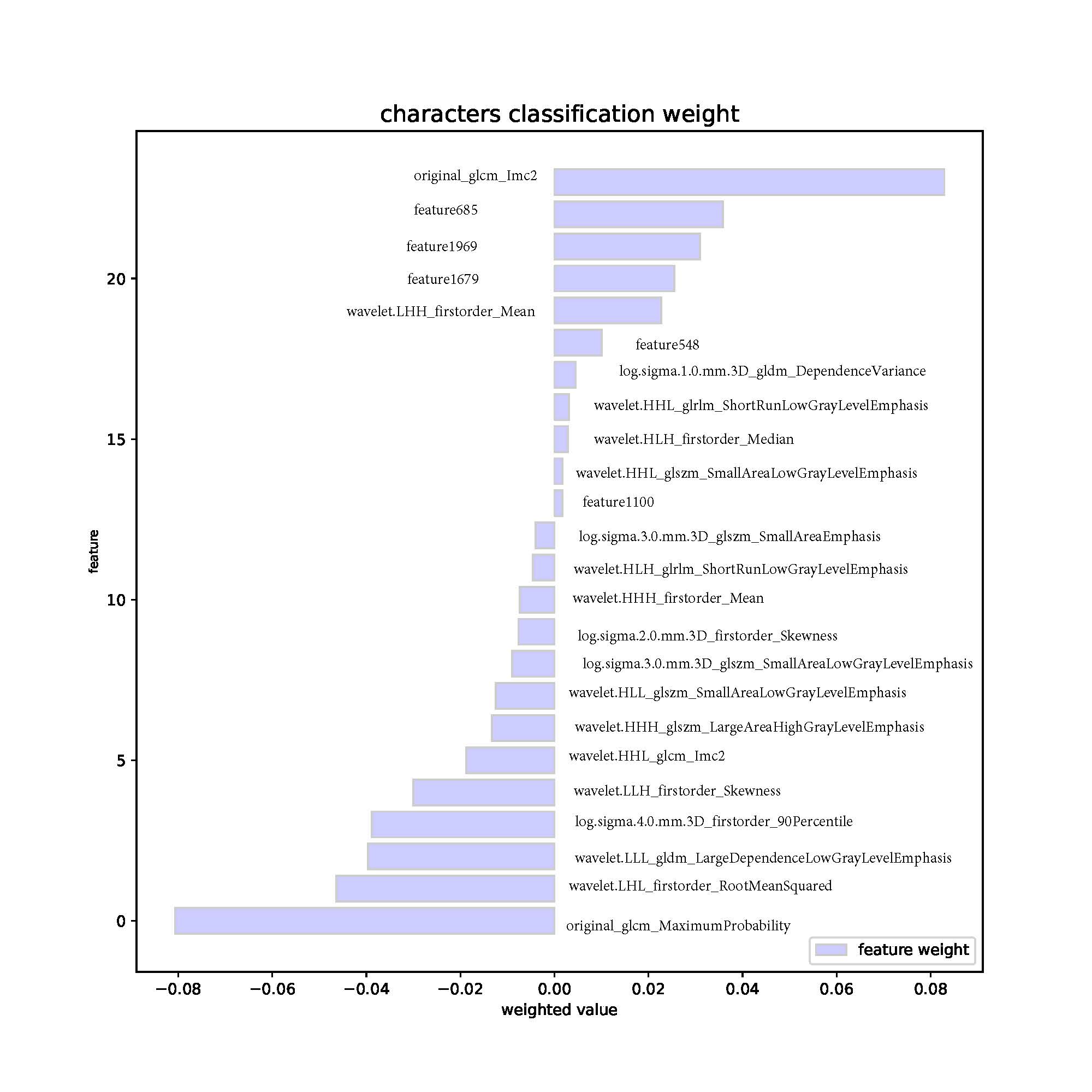


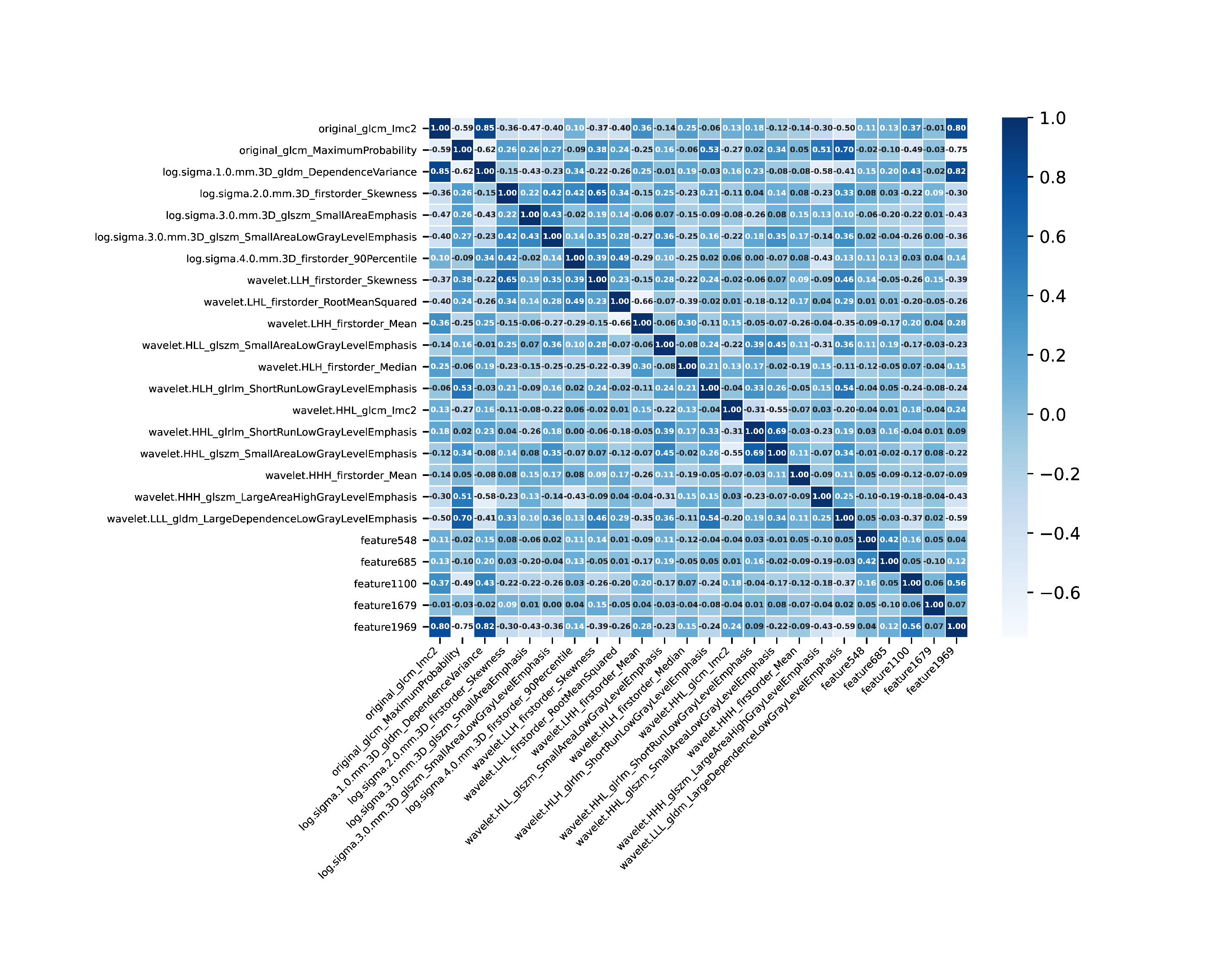
**Supplementary** **figure6:** The heatmap for selected features after LASSO regression selection and ICC analysis. 24 features are selected to create the final mode. Pearson correlation coefficients are selected for the normal distribution variables and Spearman’s correlation coefficients are selected for the non-normal variables. All variables illustrate non-strong correlations (the reference value is set to 0.9)

**Supplementary figure7:** The confusion matrix for four machine learning model and Bosniak-classification in testing datasets


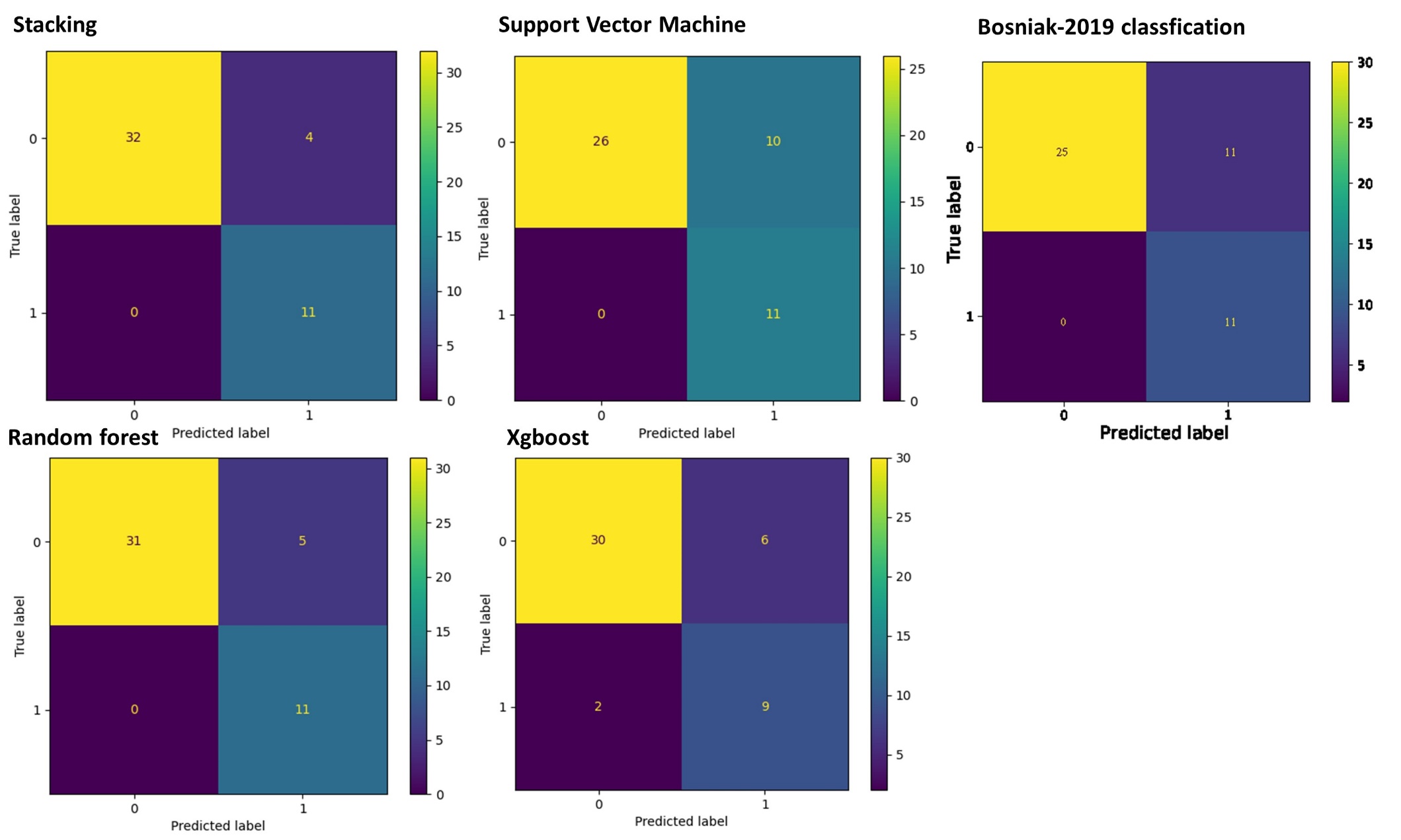


**Supplementary figure8:** The ROC curve in the training cohort using 5-fold cross-validation


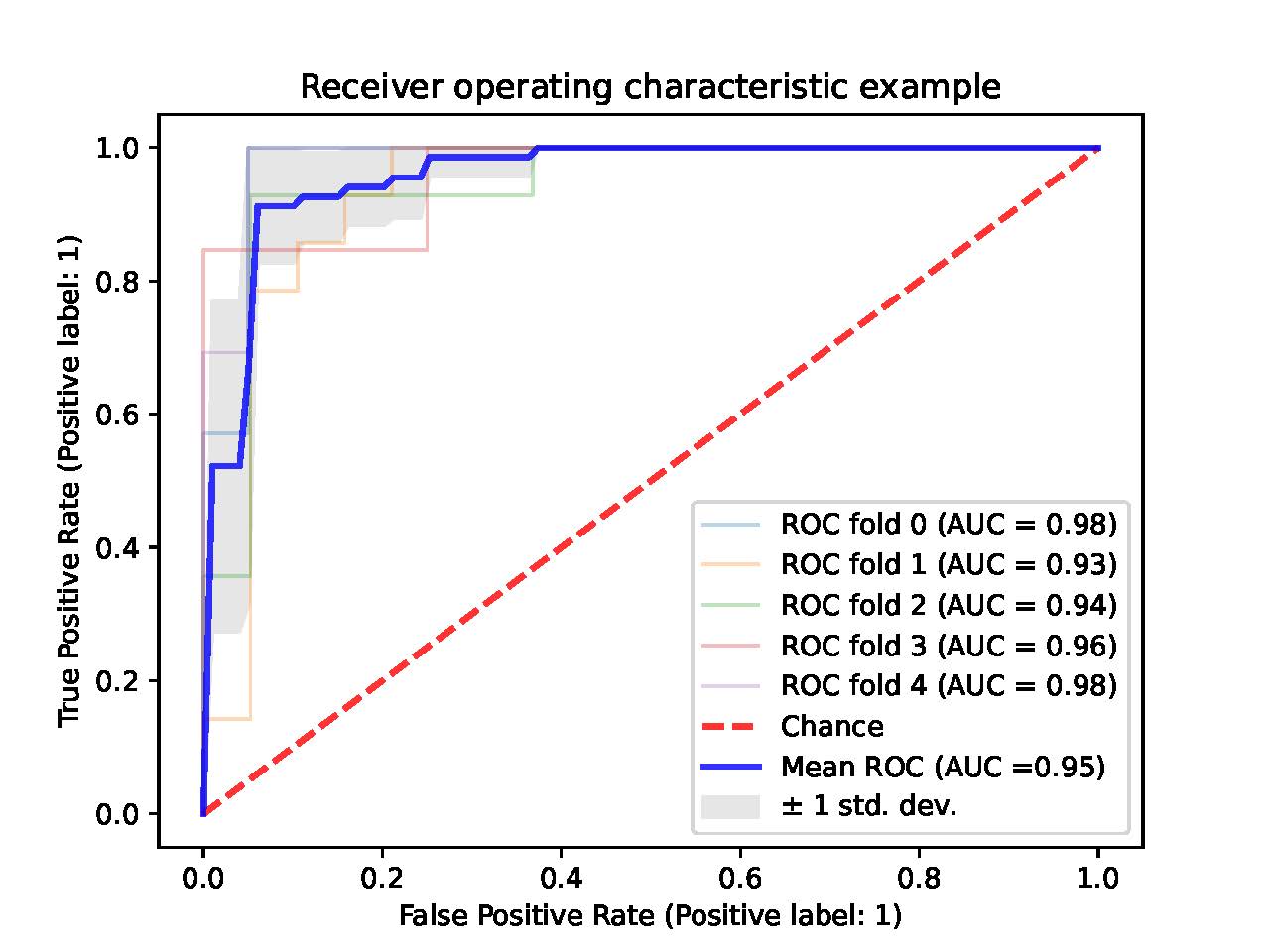


**Supplementary figure9:** Circus plot illustrating the corresponding relationship between radiomics features and 3DResnet50 features after lasso selection. wavelet.LLL_glcm_Imc2 feature and original_glcm_Imc2 demonstrate strong associations (Pearson correlation coefficients =0.99)


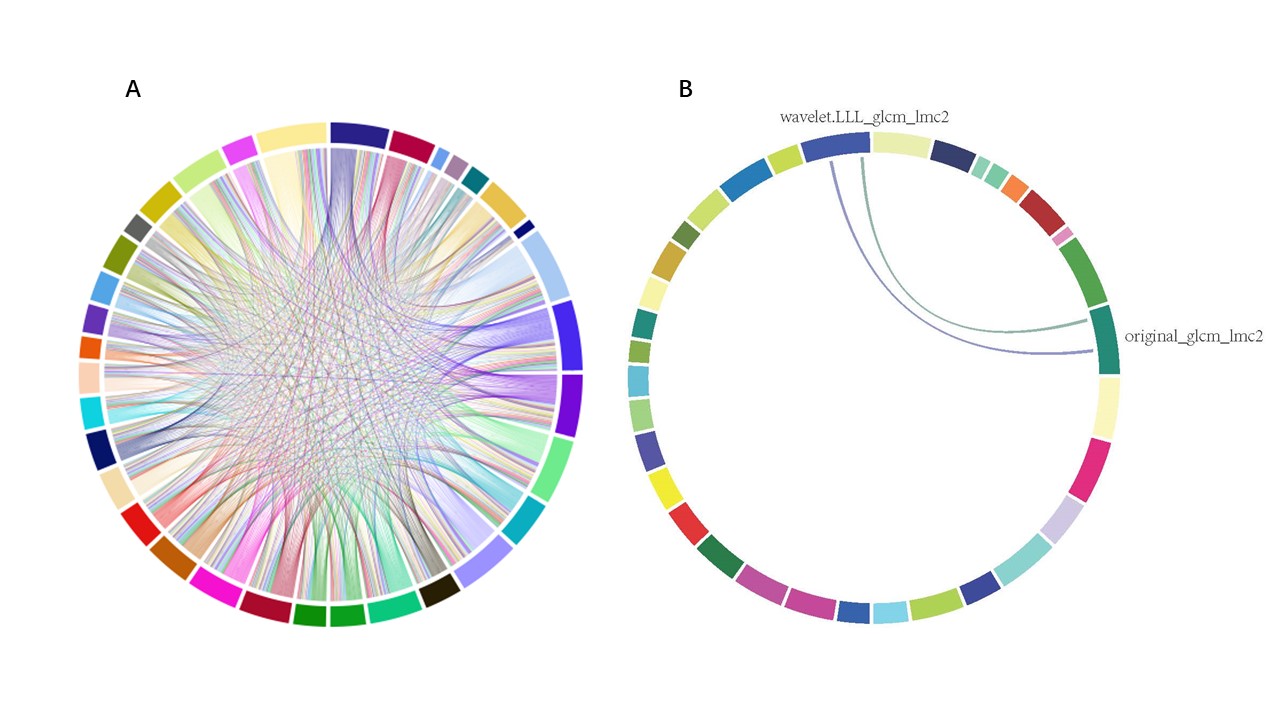


Supplementary table1: Detailed structure components and parameters in 3D-resnet50 model

| **3DResnet model structure** | | | | | |
| --- | --- | --- | --- | --- | --- |
| **Layer (type)** | **Output shape** | **Parameters** | **Layer (type)** | **Output Shape** | **Parameters** |
| Conv3d-1 | [-1, 64, 7, 64, 64] | 21,952 | BatchNorm3d-88 | [-1, 1024, 2, 16, 16] | 2,048 |
| BatchNorm3d-2 | [-1, 64, 7, 64, 64] | 128 | ReLU-89 | [-1, 1024, 2, 16, 16] | 0 |
| ReLU-3 | [-1, 64, 7, 64, 64] | 0 | Bottleneck-90 | [-1, 1024, 2, 16, 16] | 0 |
| MaxPool3d-4 | [-1, 64, 4, 32, 32] | 0 | Conv3d-91 | [-1, 256, 2, 16, 16] | 262,144 |
| Conv3d-5 | [-1, 64, 4, 32, 32] | 4,096 | BatchNorm3d-92 | [-1, 256, 2, 16, 16] | 512 |
| BatchNorm3d-6 | [-1, 64, 4, 32, 32] | 128 | ReLU-93 | [-1, 256, 2, 16, 16] | 0 |
| ReLU-7 | [-1, 64, 4, 32, 32] | 0 | Conv3d-94 | [-1, 256, 2, 16, 16] | 1,769,472 |
| Conv3d-8 | [-1, 64, 4, 32, 32] | 110,592 | BatchNorm3d-95 | [-1, 256, 2, 16, 16] | 512 |
| BatchNorm3d-9 | [-1, 64, 4, 32, 32] | 128 | ReLU-96 | [-1, 256, 2, 16, 16] | 0 |
| ReLU-10 | [-1, 64, 4, 32, 32] | 0 | Conv3d-97 | [-1, 1024, 2, 16, 16] | 262,144 |
| Conv3d-11 | [-1, 256, 4, 32, 32] | 16,384 | BatchNorm3d-98 | [-1, 1024, 2, 16, 16] | 2,048 |
| BatchNorm3d-12 | [-1, 256, 4, 32, 32] | 512 | ReLU-99 | [-1, 1024, 2, 16, 16] | 0 |
| Conv3d-13 | [-1, 256, 4, 32, 32] | 16,384 | Bottleneck-100 | [-1, 1024, 2, 16, 16] | 0 |
| BatchNorm3d-14 | [-1, 256, 4, 32, 32] | 512 | Conv3d-101 | [-1, 256, 2, 16, 16] | 262,144 |
| ReLU-15 | [-1, 256, 4, 32, 32] | 0 | BatchNorm3d-102 | [-1, 256, 2, 16, 16] | 512 |
| Bottleneck-16 | [-1, 256, 4, 32, 32] | 0 | ReLU-103 | [-1, 256, 2, 16, 16] | 0 |
| Conv3d-17 | [-1, 64, 4, 32, 32] | 16,384 | Conv3d-104 | [-1, 256, 2, 16, 16] | 1,769,472 |
| BatchNorm3d-18 | [-1, 64, 4, 32, 32] | 128 | BatchNorm3d-105 | [-1, 256, 2, 16, 16] | 512 |
| ReLU-19 | [-1, 64, 4, 32, 32] | 0 | ReLU-106 | [-1, 256, 2, 16, 16] | 0 |
| Conv3d-20 | [-1, 64, 4, 32, 32] | 110,592 | Conv3d-107 | [-1, 1024, 2, 16, 16] | 262,144 |
| BatchNorm3d-21 | [-1, 64, 4, 32, 32] | 128 | BatchNorm3d-108 | [-1, 1024, 2, 16, 16] | 2,048 |
| ReLU-22 | [-1, 64, 4, 32, 32] | 0 | ReLU-109 | [-1, 1024, 2, 16, 16] | 0 |
| Conv3d-23 | [-1, 256, 4, 32, 32] | 16,384 | Bottleneck-110 | [-1, 1024, 2, 16, 16] | 0 |
| BatchNorm3d-24 | [-1, 256, 4, 32, 32] | 512 | Conv3d-111 | [-1, 256, 2, 16, 16] | 262,144 |
| ReLU-25 | [-1, 256, 4, 32, 32] | 0 | BatchNorm3d-112 | [-1, 256, 2, 16, 16] | 512 |
| Bottleneck-26 | [-1, 256, 4, 32, 32] | 0 | ReLU-113 | [-1, 256, 2, 16, 16] | 0 |
| Conv3d-27 | [-1, 64, 4, 32, 32] | 16,384 | Conv3d-114 | [-1, 256, 2, 16, 16] | 1,769,472 |
| BatchNorm3d-28 | [-1, 64, 4, 32, 32] | 128 | BatchNorm3d-115 | [-1, 256, 2, 16, 16] | 512 |
| ReLU-29 | [-1, 64, 4, 32, 32] | 0 | ReLU-116 | [-1, 256, 2, 16, 16] | 0 |
| Conv3d-30 | [-1, 64, 4, 32, 32] | 110,592 | Conv3d-117 | [-1, 1024, 2, 16, 16] | 262,144 |
| BatchNorm3d-31 | [-1, 64, 4, 32, 32] | 128 | BatchNorm3d-118 | [-1, 1024, 2, 16, 16] | 2,048 |
| ReLU-32 | [-1, 64, 4, 32, 32] | 0 | ReLU-119 | [-1, 1024, 2, 16, 16] | 0 |
| Conv3d-33 | [-1, 256, 4, 32, 32] | 16,384 | Bottleneck-120 | [-1, 1024, 2, 16, 16] | 0 |
| BatchNorm3d-34 | [-1, 256, 4, 32, 32] | 512 | Conv3d-121 | [-1, 256, 2, 16, 16] | 262,144 |
| ReLU-35 | [-1, 256, 4, 32, 32] | 0 | BatchNorm3d-122 | [-1, 256, 2, 16, 16] | 512 |
| Bottleneck-36 | [-1, 256, 4, 32, 32] | 0 | ReLU-123 | [-1, 256, 2, 16, 16] | 0 |
| Conv3d-37 | [-1, 128, 4, 32, 32] | 32,768 | Conv3d-124 | [-1, 256, 2, 16, 16] | 1,769,472 |
| BatchNorm3d-38 | [-1, 128, 4, 32, 32] | 256 | BatchNorm3d-125 | [-1, 256, 2, 16, 16] | 512 |
| ReLU-39 | [-1, 128, 4, 32, 32] | 0 | ReLU-126 | [-1, 256, 2, 16, 16] | 0 |
| Conv3d-40 | [-1, 128, 2, 16, 16] | 442,368 | Conv3d-127 | [-1, 1024, 2, 16, 16] | 262,144 |
| BatchNorm3d-41 | [-1, 128, 2, 16, 16] | 256 | BatchNorm3d-128 | [-1, 1024, 2, 16, 16] | 2,048 |
| ReLU-42 | [-1, 128, 2, 16, 16] | 0 | ReLU-129 | [-1, 1024, 2, 16, 16] | 0 |
| Conv3d-43 | [-1, 512, 2, 16, 16] | 65,536 | Bottleneck-130 | [-1, 1024, 2, 16, 16] | 0 |
| BatchNorm3d-44 | [-1, 512, 2, 16, 16] | 1,024 | Conv3d-131 | [-1, 256, 2, 16, 16] | 262,144 |
| Conv3d-45 | [-1, 512, 2, 16, 16] | 131,072 | BatchNorm3d-132 | [-1, 256, 2, 16, 16] | 512 |
| BatchNorm3d-46 | [-1, 512, 2, 16, 16] | 1,024 | ReLU-133 | [-1, 256, 2, 16, 16] | 0 |
| ReLU-47 | [-1, 512, 2, 16, 16] | 0 | Conv3d-134 | [-1, 256, 2, 16, 16] | 1,769,472 |
| Bottleneck-48 | [-1, 512, 2, 16, 16] | 0 | BatchNorm3d-135 | [-1, 256, 2, 16, 16] | 512 |
| Conv3d-49 | [-1, 128, 2, 16, 16] | 65,536 | ReLU-136 | [-1, 256, 2, 16, 16] | 0 |
| BatchNorm3d-50 | [-1, 128, 2, 16, 16] | 256 | Conv3d-137 | [-1, 1024, 2, 16, 16] | 262,144 |
| ReLU-51 | [-1, 128, 2, 16, 16] | 0 | BatchNorm3d-138 | [-1, 1024, 2, 16, 16] | 2,048 |
| Conv3d-52 | [-1, 128, 2, 16, 16] | 442,368 | ReLU-139 | [-1, 1024, 2, 16, 16] | 0 |
| BatchNorm3d-53 | [-1, 128, 2, 16, 16] | 256 | Bottleneck-140 | [-1, 1024, 2, 16, 16] | 0 |
| ReLU-54 | [-1, 128, 2, 16, 16] | 0 | Conv3d-141 | [-1, 512, 2, 16, 16] | 524,288 |
| Conv3d-55 | [-1, 512, 2, 16, 16] | 65,536 | BatchNorm3d-142 | [-1, 512, 2, 16, 16] | 1,024 |
| BatchNorm3d-56 | [-1, 512, 2, 16, 16] | 1,024 | ReLU-143 | [-1, 512, 2, 16, 16] | 0 |
| ReLU-57 | [-1, 512, 2, 16, 16] | 0 | Conv3d-144 | [-1, 512, 2, 16, 16] | 7,077,888 |
| Bottleneck-58 | [-1, 512, 2, 16, 16] | 0 | BatchNorm3d-145 | [-1, 512, 2, 16, 16] | 1,024 |
| Conv3d-59 | [-1, 128, 2, 16, 16] | 65,536 | ReLU-146 | [-1, 512, 2, 16, 16] | 0 |
| BatchNorm3d-60 | [-1, 128, 2, 16, 16] | 256 | Conv3d-147 | [-1, 2048, 2, 16, 16] | 1,048,576 |
| ReLU-61 | [-1, 128, 2, 16, 16] | 0 | BatchNorm3d-148 | [-1, 2048, 2, 16, 16] | 4,096 |
| Conv3d-62 | [-1, 128, 2, 16, 16] | 442,368 | Conv3d-149 | [-1, 2048, 2, 16, 16] | 2,097,152 |
| BatchNorm3d-63 | [-1, 128, 2, 16, 16] | 256 | BatchNorm3d-150 | [-1, 2048, 2, 16, 16] | 4,096 |
| ReLU-64 | [-1, 128, 2, 16, 16] | 0 | ReLU-151 | [-1, 2048, 2, 16, 16] | 0 |
| Conv3d-65 | [-1, 512, 2, 16, 16] | 65,536 | Bottleneck-152 | [-1, 2048, 2, 16, 16] | 0 |
| BatchNorm3d-66 | [-1, 512, 2, 16, 16] | 1,024 | Conv3d-153 | [-1, 512, 2, 16, 16] | 1,048,576 |
| ReLU-67 | [-1, 512, 2, 16, 16] | 0 | BatchNorm3d-154 | [-1, 512, 2, 16, 16] | 1,024 |
| Bottleneck-68 | [-1, 512, 2, 16, 16] | 0 | ReLU-155 | [-1, 512, 2, 16, 16] | 0 |
| Conv3d-69 | [-1, 128, 2, 16, 16] | 65,536 | Conv3d-156 | [-1, 512, 2, 16, 16] | 7,077,888 |
| BatchNorm3d-70 | [-1, 128, 2, 16, 16] | 256 | BatchNorm3d-157 | [-1, 512, 2, 16, 16] | 1,024 |
| ReLU-71 | [-1, 128, 2, 16, 16] | 0 | ReLU-158 | [-1, 512, 2, 16, 16] | 0 |
| Conv3d-72 | [-1, 128, 2, 16, 16] | 442,368 | Conv3d-159 | [-1, 2048, 2, 16, 16] | 1,048,576 |
| BatchNorm3d-73 | [-1, 128, 2, 16, 16] | 256 | BatchNorm3d-160 | [-1, 2048, 2, 16, 16] | 4,096 |
| ReLU-74 | [-1, 128, 2, 16, 16] | 0 | ReLU-161 | [-1, 2048, 2, 16, 16] | 0 |
| Conv3d-75 | [-1, 512, 2, 16, 16] | 65,536 | Bottleneck-162 | [-1, 2048, 2, 16, 16] | 0 |
| BatchNorm3d-76 | [-1, 512, 2, 16, 16] | 1,024 | Conv3d-163 | [-1, 512, 2, 16, 16] | 1,048,576 |
| ReLU-77 | [-1, 512, 2, 16, 16] | 0 | BatchNorm3d-164 | [-1, 512, 2, 16, 16] | 1,024 |
| Bottleneck-78 | [-1, 512, 2, 16, 16] | 0 | ReLU-165 | [-1, 512, 2, 16, 16] | 0 |
| Conv3d-79 | [-1, 256, 2, 16, 16] | 131,072 | Conv3d-166 | [-1, 512, 2, 16, 16] | 7,077,888 |
| BatchNorm3d-80 | [-1, 256, 2, 16, 16] | 512 | BatchNorm3d-167 | [-1, 512, 2, 16, 16] | 1,024 |
| ReLU-81 | [-1, 256, 2, 16, 16] | 0 | ReLU-168 | [-1, 512, 2, 16, 16] | 0 |
| Conv3d-82 | [-1, 256, 2, 16, 16] | 1,769,472 | Conv3d-169 | [-1, 2048, 2, 16, 16] | 1,048,576 |
| BatchNorm3d-83 | [-1, 256, 2, 16, 16] | 512 | BatchNorm3d-170 | [-1, 2048, 2, 16, 16] | 4,096 |
| ReLU-84 | [-1, 256, 2, 16, 16] | 0 | ReLU-171 | [-1, 2048, 2, 16, 16] | 0 |
| Conv3d-85 | [-1, 1024, 2, 16, 16] | 262,144 | Bottleneck-172 | [-1, 2048, 2, 16, 16] | 0 |
| BatchNorm3d-86 | [-1, 1024, 2, 16, 16] | 2,048 | AdaptiveMaxPool3d-173 | [-1, 2048, 1, 1, 1] | 0 |

| Conv3d-87 | [-1, 1024, 2, 16, 16] | 524,288 | ResNet-174 | [-1, 2048, 1, 1, 1] | 0 |
| --- | --- | --- | --- | --- | --- |
